# Hypervolemia induces and potentiates lung damage after recruitment maneuver in a model of sepsis-induced acute lung injury

**DOI:** 10.1186/cc9063

**Published:** 2010-06-14

**Authors:** Pedro L Silva, Fernanda F Cruz, Livia C Fujisaki, Gisele P Oliveira, Cynthia S Samary, Debora S Ornellas, Tatiana Maron-Gutierrez, Nazareth N Rocha, Regina Goldenberg, Cristiane SNB Garcia, Marcelo M Morales, Vera L Capelozzi, Marcelo Gama de Abreu, Paolo Pelosi, Patricia RM Rocco

**Affiliations:** 1 Laboratory of Pulmonary Investigation, Carlos Chagas Filho Institute of Biophysics, Federal University of Rio de Janeiro, Av. Carlos Chagas Filho, s/n, Rio de Janeiro, 21949-902, Brazil; 2 Laboratory of Cellular and Molecular Physiology, Carlos Chagas Filho Institute of Biophysics, Federal University of Rio de Janeiro, Av. Carlos Chagas Filho, s/n, Rio de Janeiro, 21949-902, Brazil; 3 Laboratory of Cell and Molecular Cardiology, Carlos Chagas Filho Institute of Biophysics, Federal University of Rio de Janeiro, Av. Carlos Chagas Filho, s/n, Rio de Janeiro, 21949-902, Brazil; 4 Department of Physiology and Pharmacology, Fluminense Federal University, Rua Professor Hernani Pires de Melo 101, Niterói, Rio de Janeiro, 24210-130, Brazil; 5 Department of Pathology, Faculty of Medicine, University of São Paulo, Dr. Arnaldo Street, 455, Sao Paulo, 01246-903, Brazil; 6 Pulmonary Engineering Group, Department of Anaesthesiology and Intensive Care Therapy, University Hospital Carl Gustav Carus, Technical University of Dresden, Fetscherstr. 74, 01307 Dresden, Germany; 7 Department of Ambient, Health and Safety, University of Insubria, Servizio di Anestesia B, Ospedale di Circolo e Fondazione Macchi viale Borri 57, 21100 Varese, Italy

## Abstract

**Introduction:**

Recruitment maneuvers (RMs) seem to be more effective in extrapulmonary acute lung injury (ALI), caused mainly by sepsis, than in pulmonary ALI. Nevertheless, the maintenance of adequate volemic status is particularly challenging in sepsis. Since the interaction between volemic status and RMs is not well established, we investigated the effects of RMs on lung and distal organs in the presence of hypovolemia, normovolemia, and hypervolemia in a model of extrapulmonary lung injury induced by sepsis.

**Methods:**

ALI was induced by cecal ligation and puncture surgery in 66 Wistar rats. After 48 h, animals were anesthetized, mechanically ventilated and randomly assigned to 3 volemic status (n = 22/group): 1) hypovolemia induced by blood drainage at mean arterial pressure (MAP)≈70 mmHg; 2) normovolemia (MAP≈100 mmHg), and 3) hypervolemia with colloid administration to achieve a MAP≈130 mmHg. In each group, animals were further randomized to be recruited (CPAP = 40 cm H_2_O for 40 s) or not (NR) (n = 11/group), followed by 1 h of protective mechanical ventilation. Echocardiography, arterial blood gases, static lung elastance (Est,L), histology (light and electron microscopy), lung wet-to-dry (W/D) ratio, interleukin (IL)-6, IL-1β, caspase-3, type III procollagen (PCIII), intercellular adhesion molecule-1 (ICAM-1), and vascular cell adhesion molecule-1 (VCAM-1) mRNA expressions in lung tissue, as well as lung and distal organ epithelial cell apoptosis were analyzed.

**Results:**

We observed that: 1) hypervolemia increased lung W/D ratio with impairment of oxygenation and Est,L, and was associated with alveolar and endothelial cell damage and increased IL-6, VCAM-1, and ICAM-1 mRNA expressions; and 2) RM reduced alveolar collapse independent of volemic status. In hypervolemic animals, RM improved oxygenation above the levels observed with the use of positive-end expiratory pressure (PEEP), but increased lung injury and led to higher inflammatory and fibrogenetic responses.

**Conclusions:**

Volemic status should be taken into account during RMs, since in this sepsis-induced ALI model hypervolemia promoted and potentiated lung injury compared to hypo- and normovolemia.

## Introduction

Recent studies have demonstrated that low tidal volume (V_T _= 6 ml/kg) significantly reduces morbidity and mortality in patients with acute lung injury/acute respiratory distress syndrome (ALI/ARDS) [1]. Such strategy requires the use of moderate-to-high positive end-expiratory pressure (PEEP) and may be combined with recruitment maneuvers (RMs) [2,3]. Although the use of RMs and high PEEP is not routinely recommended, they seem effective at improving oxygenation with minor adverse effects and should be considered for use on an individualized basis in patients with ALI/ARDS who have life-threatening hypoxemia [4]. Additionally, RMs associated with higher PEEP have been shown to reduce hypoxemia-related deaths and can be used as rescue therapies in ALI/ARDS patients [3]. However, RMs may also exacerbate epithelial [5-9] and endothelial [10] damage, increasing alveolar capillary permeability [8]. Furthermore, transient increase in intrathoracic pressure during RMs may lead to hemodynamic instability [11] and distal organ injury [12]. Despite these potential deleterious effects, RMs have been recognized as effective for improving oxygenation, at least transiently [4] and even reducing the need for rescue therapies in severe hypoxemia [3]. To minimize hemodynamic instability associated with RMs, the use of fluids has been described [13]. However, fluid management itself may have an impact on lung and distal organ injury in ALI/ARDS [14,15]. Although fluid restriction may cause distal organ damage [14], hypervolemia has been associated with increased lung injury [16,17].

RMs seem to be more effective in extrapulmonary ALI/ARDS [9], caused mainly by sepsis [18], than in pulmonary ALI/ARDS. Nevertheless, the maintenance of adequate volemic status is particularly challenging in sepsis. As the interaction between volemic status and RMs is not well established, we hypothesized that volemic status would potentiate possible deleterious effects of RMs on lung and distal organs in a model of extrapulmonary lung injury induced by sepsis. Therefore, we compared the effects of RMs in the presence of hypovolemia, normovolemia, and hypervolemia on arterial blood gases, static lung elastance (Est,L), histology (light and electron microscopy), lung wet-to-dry (W/D) ratio, IL-6, IL-1β, caspase-3, type III procollagen (PCIII), intercellular adhesion molecule 1 (ICAM-1), and vascular cell adhesion molecule 1 (VCAM-1) mRNA expressions in lung tissue, as well as lung and distal organ epithelial cell apoptosis in an experimental model of sepsis-induced ALI.

## Materials and methods

### Animal preparation and experimental protocol

This study was approved by the Ethics Committee of the Health Sciences Center, Federal University of Rio de Janeiro. All animals received humane care in compliance with the *Principles of Laboratory Animal Care *formulated by the National Society for Medical Research and the *Guide for the Care and Use of Laboratory Animals *prepared by the National Academy of Sciences, USA.

Sixty-six adult male Wistar rats (270 to 300 g) were kept under specific pathogen-free conditions in the animal care facility at the Laboratory of Pulmonary Investigation, Federal University of Rio de Janeiro. In 36 rats, Est,L, histology, and molecular biology were analyzed. The remaining 30 rats were used to evaluate lung W/D ratio. Animals were fasted for 16 hours before the surgical procedure. Following that, sepsis was induced by cecal ligation and puncture (CLP) as described in previous studies [19]. Briefly, animals were anesthetized with sevoflurane and a midline laparotomy (2 cm incision) was performed. The cecum was carefully isolated to avoid damage to blood vessels, and a 3.0 cotton ligature was placed below the ileocecal valve to prevent bowel obstruction. Finally, the cecum was punctured twice with an 18 gauge needle [20] and animals recovered from anesthesia. Soon after surgery, each rat received a subcutaneous injection of 1 ml of warm (37°C) normal saline with tramadol hydrochloride (20 μg/g body weight).

**Figure 1:**
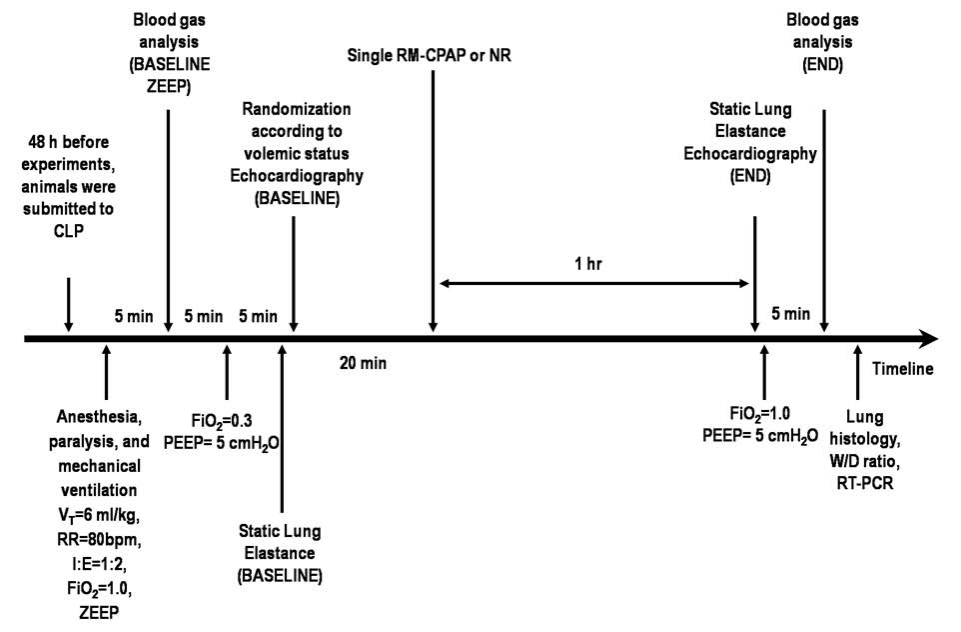
**Timeline representation of the experimental protocol**. CLP, cecal ligation and puncture; I:E, inspiratory-to-expiratory ratio; PEEP, positive end-expiratory pressure; RR, respiratory rate; RT-PCR, real time-polymerase chain reaction; V_T_, tidal volume; W/D ratio, lung wet-to-dry ratio; ZEEP, zero end-expiratory pressure.

Figure 1 depicts the time-course of interventions. Forty-eight hours after surgery, rats were sedated (diazepam 5 mg intraperitoneally), anesthetized (thiopental sodium 20 mg/kg intraperitoneally), tracheotomized, and a polyethylene catheter (PE-10; SCIREQ, Montreal, Canada) was introduced into the carotid artery for blood sampling and monitoring of mean arterial pressure (MAP). The animals were then paralyzed (vecuronium bromide 2 mg/kg, intravenously) and mechanically ventilated (Servo i, MAQUET, Switzerland) with the following parameters: V_T _= 6 ml/kg, respiratory rate (RR) = 80 breaths/min, inspiratory to expiratory ratio = 1:2, fraction of inspired oxygen (FiO_2_) = 1.0, and PEEP equal to 0 cmH_2_O (zero end-expiratory pressure (ZEEP)). Blood (300 μl) was drawn into a heparinized syringe for measurement of arterial oxygen partial pressure (PaO_2_), arterial carbon dioxide partial pressure (PaCO_2_) and arterial pH (pHa) (i-STAT, Abbott Laboratories, North Chicago, IL, USA) (BASELINE-ZEEP). Afterwards, mechanical ventilation was set according to the following parameters: V_T _= 6 ml/kg, RR = 80 bpm, PEEP = 5 cmH_2_O, and FiO_2 _= 0.3 (Figure 1). Est,L was then measured (BASELINE) and the animals were randomly assigned to one of the following groups: 1) hypovolemia (HYPO); 2) normovolemia (NORMO), and 3) hypervolemia (HYPER). Hypovolemia was induced by blood drainage in order to achieve a MAP of about 70 mmHg. Normovolemia was maintained at a MAP of about 100 mmHg. Hypervolemia was obtained with colloid administration (Gelafundin^®^; B. Braun, Melsungen, Germany) at an infusion rate of 2 ml/kg/min to achieve a MAP of about 130 mmHg. Following that, the colloid infusion rate was reduced to 1 ml/kg/min in order to maintain a constant MAP. Depth of anesthesia was similar in all animals and a comparable amount of sedative and anesthetic drugs were given in all groups. After achieving volemic status, animals were further randomized to be recruited, with a single RM consisting of continuous positive airway pressure (CPAP) of 40 cmH_2_O for 40 seconds (RM-CPAP), or not (NR) (n = 6 per group; Figure 1). After one hour of mechanical ventilation (END), Est,L was measured. FiO_2 _was then increased to 1.0, and after five minutes arterial blood gases were analyzed (END). Finally, the animals were euthanized and lungs, kidney, liver and small intestine were prepared for histology. IL-6, IL-1β, caspase-3, and PCIII mRNA expressions were measured in lung tissue. The experiments took no longer than 80 minutes.

### Respiratory parameters

Airflow, airway and esophageal pressures were measured [9,21]. Changes in esophageal pressure, which reflect chest wall pressure, were measured with a water-filled catheter (PE205) with side holes at the tip connected to a SCIREQ differential pressure transducer (SC-24, Montreal, Canada). Before animals were paralyzed, the catheter was passed into the stomach, slowly returned into the esophagus, and its proper positioning was assessed using the 'occlusion test' [22,23]. Transpulmonary pressure was calculated by the difference between airway and esophageal pressures [9]. All signals were filtered (100 Hz), amplified in a four-channel conditioner (SC-24, SCIREQ, Montreal, Quebec, Canada), sampled at 200 Hz with a 12-bit analogue-to-digital converter (DT2801A, Data Translation, Marlboro, MA, USA) and continuously recorded throughout the experiment by a personal computer. To calculate Est,L, airways were occluded at end-inspiration until a transpulmonary plateau pressure was reached (at the end of five seconds), after which this value was divided by V_T _[9,21]. All data were analyzed using ANADAT data analysis software (RHT-InfoData, Inc., Montreal, Quebec, Canada).

### Echocardiography

Volemic status and cardiac function were assessed by an echocardiograph equipped with a 10 MHz mechanical transducer (Esaote model, CarisPlus, Firenze, Italy). Images were obtained from the subcostal and parasternal views. Short-axis B-dimensional views of the left ventricle were acquired at the level of the papillary muscles to obtain the M-mode image. The inferior vena cava (IVC) and right atrium (RA) diameters were measured from the subcostal approach. Cardiac output, stroke volume, and ejection fraction were obtained from the B-mode according to Simpson's method [24].

### Light microscopy

A laparotomy was performed immediately after determination of lung mechanics and heparin (1,000 IU) was intravenously injected in the vena cava. The trachea was clamped at end-expiration (PEEP = 5 cmH_2_0), and the abdominal aorta and vena cava were sectioned, yielding a massive hemorrhage that quickly killed the animals. Right lung, kidney, liver, and small intestine were then removed, fixed in 3% buffered formaldehyde and paraffin-embedded. Four-μm-thick slices were cut and stained with H&E.

Lung morphometric analysis was performed using an integrating eyepiece with a coherent system consisting of a grid with 100 points and 50 lines (known length) coupled to a conventional light microscope (Olympus BX51, Olympus Latin America-Inc., São Paulo, Brazil). The volume fraction of the lung occupied by collapsed alveoli or normal pulmonary areas or hyperinflated structures (alveolar ducts, alveolar sacs, or alveoli, all wider than 120 μm) was determined by the point-counting technique [25] at a magnification of × 200 across 10 random, non-coincident microscopic fields [26].

### Transmission electron microscopy

Three slices measuring 2 × 2 × 2 mm were cut from three different segments of the left lung and fixed (2.5% glutaraldehyde and phosphate buffer 0.1 M (pH = 7.4)) for electron microscopy (JEOL 1010 Transmission Electron Microscope, Tokyo, Japan) analysis. For each electron microscopy image (15 per animal), the following structural damages were analyzed: a) alveolar capillary membrane, b) type II epithelial cells, and c) endothelial cells. Pathologic findings were graded according to a five-point semi-quantitative severity-based scoring system as: 0 = normal lung parenchyma, 1 = changes in 1 to 25%, 2 = changes in 26 to 50%, 3 = changes in 51 to 75%, and 4 = changes in 76 to 100% of examined tissue [9,21].

### Apoptosis assay of lung, kidney, liver and small intestine villi

Terminal deoxynucleotidyl transferase biotin-dUTP nick end labeling (TUNEL) staining was used in a blinded fashion by two pathologists to assay cellular apoptosis. Apoptotic cells were detected using In Situ Cell Death Detection Kit, Fluorescin (Boehringer, Mannheim, Frankfurt, Germany). The nuclei without DNA fragmentation stained blue as a result of counterstaining with hematoxylin [20]. Ten fields per section from the regions with apoptotic cells were examined at a magnification of × 400. A five-point semi-quantitative severity-based scoring system was used to assess the degree of apoptosis, graded as: 0 = normal lung parenchyma; 1 = 1-25%; 2 = 26 to 50%; 3 = 51 to 75%; and 4 = 76 to 100% of examined tissue.

### IL-6, IL-1β, caspase-3, PCIII, VCAM-1, and ICAM-1 mRNA expressions

Quantitative real-time RT-PCR was performed to measure the expression of IL-6, IL-1β, caspase-3, PCIII, VCAM, and ICAM genes. Central slices of left lung were cut, collected in cryotubes, quick-frozen by immersion in liquid nitrogen and stored at -80°C. Total RNA was extracted from the frozen tissues using Trizol reagent (Invitrogen, Carlsbad, CA, USA) according to manufacturer's recommendations. RNA concentration was measured by spectrophotometry in Nanodrop^® ^ND-1000 (Thermo Fisher Scientific, Wilmington, DE, USA). First-strand cDNA was synthesized from total RNA using M-MLV Reverse Transcriptase Kit (Invitrogen, Carlsbad, CA, USA). PCR primers for target gene were purchased (Invitrogen, Carlsbad, CA, USA). The following primers were used: IL-1β (sense 5'-CTA TGT CTT GCC CGT GGA G-3', and antisense 5'-CAT CAT CCC ACG AGT CAC A-3'); IL- 6 (sense 5'-CTC CGC AAG AGA CTT CCA G-3' and antisense 5'-CTC CTC TCC GGA CTT GTG A-3'); PCIII (sense 5'-ACC TGG ACC ACA AGG ACA C-3' and antisense 5'-TGG ACC CAT TTC ACC TTT C-3'); caspase-3 (sense 5'-GGC CGA CTT CCT GTA TGC-3' and antisense 5'-GCG CAA AGT GAC TGG ATG-3'); VCAM-1 (sense 5'-TGC ACG GTC CCT AAT GTG TA-3' and antisense 5'-TGC CAA TTT CCT CCC TTA AA-3'); ICAM-1 (sense 5'-CTT CCG ACT AGG GTC CTG AA-3' and antisense 5'-CTT CAG AGG CAG GAA ACA GG-3'); and glyceraldehyde-3-phosphate dehydrogenase (GAPDH; sense 5'-GGT GAA GGT CGG TGTG AAC- 3' and antisense 5'-CGT TGA TGG CAA CAA TGT C-3'). Relative mRNA levels were measured with a SYBR green detection system using ABI 7500 Real-Time PCR (Applied Biosystems, Foster City, CA, USA). All samples were measured in triplicate. The relative expression of each gene was calculated as a ratio compared with the reference gene, GAPDH and expressed as fold change relative to NORMO-NR.

### Lung wet-to-dry ratio

W/D ratio was determined in the right lung as previously described [27]. Briefly, the right lung was separated, weighed (wet weight) and then dried in a microwave at low power (200 W) for five minutes. The drying process was repeated until the difference between the two consecutive lung weight measurements was less than 0.002 g. The last weight measurement represented the dry weight.

### Statistical analysis

Normality of data was tested using the Kolmogorov-Smirnov test with Lilliefors' correction, while the Levene median test was used to evaluate the homogeneity of variances. If both conditions were satisfied, one-way analysis of variance (ANOVA) for repeated measures was used to compare the time course of MAP, IVC and RA dimensions. To compare arterial blood gases, Est,L, and echocardiographic data at BASELINE and after one hour of mechanical ventilation (END), the paired *t*-test was used. Lung mechanics (END) and morphometry, echocardiographic data (END), arterial blood gases (END), W/D ratio, and inflammatory and fibrogenic mediators were analyzed using two-way ANOVA followed by Tukey's test. To compare non-parametric data, two-way ANOVA on ranks followed by Dunn's *post-hoc *test was selected. The relations between functional and morphological data were investigated with the Spearman correlation test. Parametric data were expressed as mean ± standard error of the mean, while non-parametric data were expressed as median (interquartile range). All tests were performed using the SigmaStat 3.1 statistical software package (Jandel Corporation, San Raphael, CA, USA), and statistical significance was established as *P *< 0.05.

## Results

The present CLP model of sepsis resulted in a survival rate of approximately 60% at 48 hours. No animals died during the investigation period.

In the HYPO, NORMO and HYPER groups, MAP was stabilized at 70 ± 10, 100 ± 10, and 130 ± 10 mmHg, respectively (Table 1). The smallest RA and IVC diameters were observed in the HYPO and the largest in the HYPER groups (Table 1). Stroke volume and cardiac output, as well as ejection fraction were similar at BASELINE in all groups (Table 2). In the HYPER group, stroke volume, cardiac output, and ejection fraction were increased compared with the NORMO and HYPO groups, with no significant changes after RM (Table 2).

**Table 1:** Mean arterial pressure and inferior vena cava and right atrium dimensions

			BASELINE	5 min	10 min	15 min	20 min	80 min
MAP (mmHg)	HYPO	NR	110 ± 6	107 ± 5	77 ± 4*	70 ± 3*	67 ± 3*	62 ± 3*
		RM-CPAP	110 ± 2	97 ± 2	76 ± 2*	71 ± 1*	65 ± 2*	63 ± 1*
								
	NORMO	NR	104 ± 8	101 ± 6	100 ± 6**	103 ± 6**	100 ± 4**	97 ± 4**
		RM-CPAP	103 ± 2	103 ± 2	100 ± 2‡	105 ± 3‡	96 ± 3‡	95 ± 2‡
								
	HYPER	NR	106 ± 3	128 ± 2* **#	130 ± 2* **#	131 ± 3* **#	131 ± 2* **#	126 ± 2* **#
		RM-CPAP	103 ± 2	126 ± 5*‡§	129 ± 4*‡§	128 ± 4*‡§	124 ± 2*‡§	117 ± 5*‡§

IVC (mm)	HYPO	NR	1.6 ± 0.2	1.5 ± 0.1	1.2 ± 0.1*	1.0 ± 0.1*	1.0 ± 0.1*	0.9 ± 0.0*
		RM-CPAP	1.6 ± 0.2	1.4 ± 0.1	1.1 ± 0.1*	0.9 ± 0.1*	0.8 ± 0.0*	0.7 ± 0.0*
								
	NORMO	NR	1.6 ± 0.1	1.7 ± 0.1	1.6 ± 0.1	1.7 ± 0.0**	1.7 ± 0.0**	1.5 ± 0.0**
		RM-CPAP	1.5 ± 0.0	1.5 ± 0.0	1.4 ± 0.0	1.6 ± 0.0‡	1.6 ± 0.0‡	1.4 ± 0.0‡
								
	HYPER	NR	1.4 ± 0.0	2.3 ± 0.2* **#	2.6 ± 0.1* **#	2.5 ± 0.3* **#	2.6 ± 0.3* **#	2.6 ± 0.1* **#
		RM-CPAP	1.4 ± 0.0	2.1 ± 0.2* ‡§	2.5 ± 0.1* ‡§	2.6 ± 0.1* ‡§	2.6 ± 0.2* ‡§	2.4 ± 0.2* ‡§

RA (mm)	HYPO	NR	4.0 ± 0.4	3.9 ± 0.6	3.8 ± 0.4	2.8 ± 0.2*	2.3 ± 0.3*	2.7 ± 0.2*
		RM-CPAP	4.2 ± 0.1	3.4 ± 0.1	3.1 ± 0.0*	2.9 ± 0.0*	2.5 ± 0.2*	3.0 ± 0.0*
								
	NORMO	NR	3.5 ± 0.0	3.5 ± 0.0	3.7 ± 0.0	3.5 ± 0.0**	3.6 ± 0.0**	3.3 ± 0.0**
		RM-CPAP	3.6 ± 0.1	3.5 ± 0.1	3.6 ± 0.0	3.5 ± 0.0‡	3.6 ± 0.0‡	3.5 ± 0.1‡
								
	HYPER	NR	3.9 ± 0.1	4.8 ± 0.5	6.1 ± 0.4* **#	6.5 ± 0.4* **#	7.1 ± 0.4* **#	7.4 ± 0.0* **#
		RM-CPAP	4.1 ± 0.1	6.5 ± 0.5*‡§	7.2 ± 0.3*‡§	7.2 ± 0.3*‡§	7.3 ± 0.3*‡§	7.1 ± 0.2*‡§

Mean arterial pressure (MAP), and inferior vena cava (IVC) and right atrium (RA) dimensions at BASELINE, during the induction of hyper or hypovolemia (BASELINE until 20 min), and at the end of the experiment (80 min). Animals were randomly assigned to hypovolemia (HYPO), normovolemia (NORMO) or hypervolemia (HYPER) with recruitment maneuver (RM-CPAP) or not (NR). Values are shown as mean ± standard error of the mean of six rats in each group. *Significantly different from BASELINE (*P *< 0.05). †Significantly different from NR (*P *<0.05). **Significantly different from HYPO-NR (*P *< 0.05). ‡ Significantly different from HYPO-RM-CPAP (*P *< 0.05). #Significantly different from NORMO-NR (*P *< 0.05). §Significantly different from NORMO-RM-CPAP (*P *< 0.05).

**Table 2:** Echocardiographic data

		HYPO		NORMO		HYPER	
				
		NR	RM-CPAP	NR	RM-CPAP	NR	RM-CPAP
Cardiac Output (ml.min^-1^)	BASELINE	20 ± 10	20 ± 10	20 ± 10	20 ± 10	20 ± 10	40 ± 10†§
	END	10 ± 10	10 ± 10	10 ± 10	20 ± 10	60 ± 10* **#	60 ± 10‡§

Stroke volume (ml)	BASELINE	0.17 ± 0.01	0.13 ± 0.01†	0.13 ± 0.01**	0.13 ± 0.01	0.10 ± 0.05**	0.13 ± 0.01
	END	0.10 ± 0.01*	0.10 ± 0.01	0.10 ± 0.01	0.13 ± 0.01	0.33 ± 0.01**#	0.26 ± 0.01*†‡§

Ejection fraction (%)	BASELINE	74 ± 1	73 ± 3	78 ± 4	74 ± 4	74 ± 1	68 ± 7
	END	63 ± 4*	65 ± 1*	71 ± 1	73 ± 1‡	86 ± 3* **#	88 ± 3*‡§

Echocardiographic data measured at BASELINE and after one hour of mechanical ventilation (END). Animals were randomly assigned to hypovolemia (HYPO), normovolemia (NORMO) or hypervolemia (HYPER) with recruitment maneuver (RM-CPAP) or not (NR). Values are mean ± standard error of the mean of six rats in each group. *Significantly different from BASELINE (*P *< 0.05). †Significantly different from NR (*P *< 0.05). **Significantly different from HYPO-NR (*P *< 0.05). ‡Significantly different from HYPO-RM-CPAP (*P *< 0.05). #Significantly different from NORMO-NR (*P *< 0.05). §Significantly different from NORMO-RM-CPAP (*P *< 0.05).

Table 3 shows arterial blood gases and lung mechanics in the three groups. PaO_2_, PaCO_2_, and pHa were comparable at BASELINE ZEEP in all groups. At END, PaO_2 _was lower in HYPER compared with the HYPO and NORMO groups when RMs were not applied. When RMs were applied, PaO_2 _was higher in NORMO compared with the HYPER group. In HYPER group, PaO_2 _was higher in RM-CPAP compared with the NR subgroup, while no differences in PaO_2 _were found between RM-CPAP and NR in HYPO and NORMO groups. PaCO_2 _and pHa did not change significantly in either NR or RM-CPAP regardless of volemic status. Est,L was similar at BASELINE in all groups. At END, Est,L was significantly increased in HYPER compared with HYPO and NORMO groups when RMs were not applied. Est,L was reduced in both HYPO and HYPER groups when lungs were recruited. However, Est,L did not change in NORMO group after RMs.

**Table 3:** Arterial blood gases and static lung elastance

		HYPO		NORMO		HYPER	
				
		NR	RM-CPAP	NR	RM-CPAP	NR	RM-CPAP
PaO_2_ (mmHg)	BASELINE ZEEP	225 ± 96	190 ± 38	164 ± 40	228 ± 114	147 ± 64	212 ± 88
	END	466 ± 32*	430 ± 69*	485 ± 45*	537 ± 40*	231 ± 20**#	380 ± 42†§

PaCO_2 _(mmHg)	BASELINE ZEEP	31 ± 2	30 ± 7	34 ± 4	37 ± 5	35 ± 3	37 ± 7
	END	34 ± 6	32 ± 5	28 ± 9	37 ± 3	39 ± 12	35 ± 11

pHa	BASELINE ZEEP	7.30 ± 0.10	7.23 ± 0.01	7.27 ± 0.10	7.25 ± 0.10	7.24 ± 0.10	7.22 ± 0.01
	END	7.11 ± 0.10	7.13 ± 0.01	7.19 ± 0.10	7.21 ± 0.10	7.23 ± 0.10	7.22 ± 0.01

Est,L (cmH_2_O.ml^-1^)	BASELINE	3.4 ± 0.3	3.2 ± 0.5	3.0 ± 0.3	3.1 ± 0.3	3.3 ± 0.5	3.3 ± 0.5
	END	3.1 ± 0.4	1.2 ± 0.1*†	2.6 ± 0.1	2.5 ± 0.4‡	4.1 ± 0.7#‡	2.8 ± 0.6†

Arterial oxygen partial pressure (PaO_2_, mmHg), arterial carbon dioxide partial pressure (PaCO_2_), and arterial pH (pHa) measured at BASELINE-ZEEP and after one hour of mechanical ventilation (END). Static lung elastance (Est,L) measured at BASELINE (positive end-expiratory pressure = 5 cmH_2_O) and at END. Animals were randomly assigned to hypovolemia (HYPO), normovolemia (NORMO) or hypervolemia (HYPER) with recruitment maneuver (RM-CPAP) or not (NR). Values are mean ± standard error of the mean of six rats in each group. *Significantly different from BASELINE (*P *< 0.05). †Significantly different from NR (*P *< 0.05). **Significantly different from HYPO-NR (*P *< 0.05). ‡Significantly different from HYPO-RM-CPAP (*P *< 0.05). #Significantly different from NORMO-NR (*P *< 0.05). §Significantly different from NORMO-RM-CPAP (*P *< 0.05).

**Figure 2:**
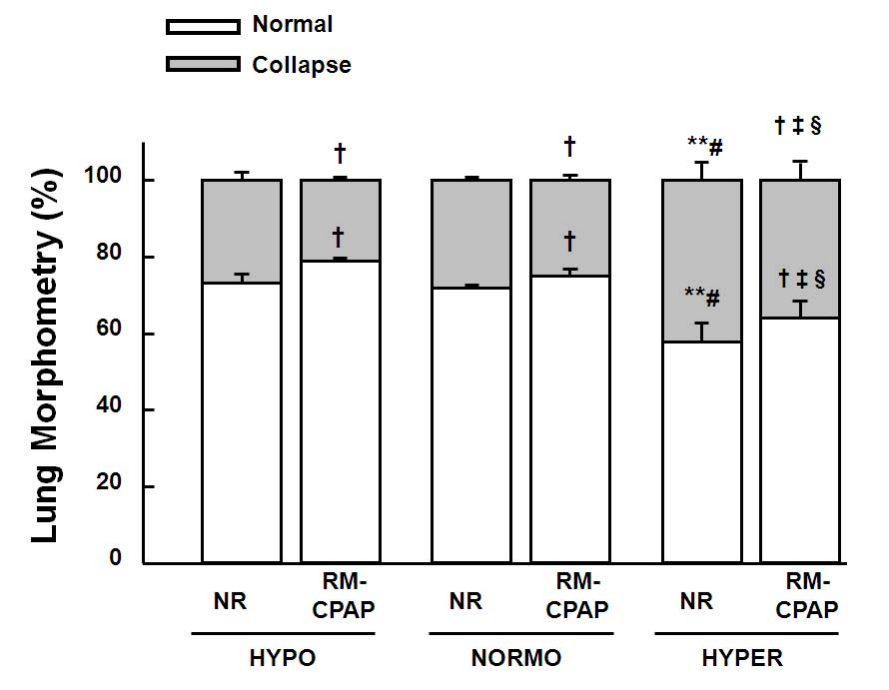
**Volume fraction of the lung occupied by collapsed alveoli (gray) or normal pulmonary areas (white)**. Animals were randomly assigned to hypovolemia (HYPO), normovolemia (NORMO) or hypervolemia (HYPER) with recruitment maneuver (RM-CPAP) or not (NR). All values were computed in 10 random, noncoincident fields per rat. Values are mean ± standard error of the mean of six animals in each group. †Significantly different from NR (*P *< 0.05). **Significantly different from HYPO-NR (*P *< 0.05). ‡Significantly different from HYPO-RM-CPAP (*P *< 0.05). #Significantly different from NORMO-NR (*P *< 0.05). §Significantly different from NORMO-RM-CPAP (*P *< 0.05).

The fraction of alveolar collapse was higher in HYPER (42%) compared with HYPO (27%) and NORMO (28%) groups. RMs decreased alveolar collapse independently of volemic status; nevertheless, alveolar collapse was more frequent in HYPER (26%) than NORMO (17%) and HYPO (12%) groups. Hyperinflated areas were not detected in any group (Figure 2).

**Figure 3:**
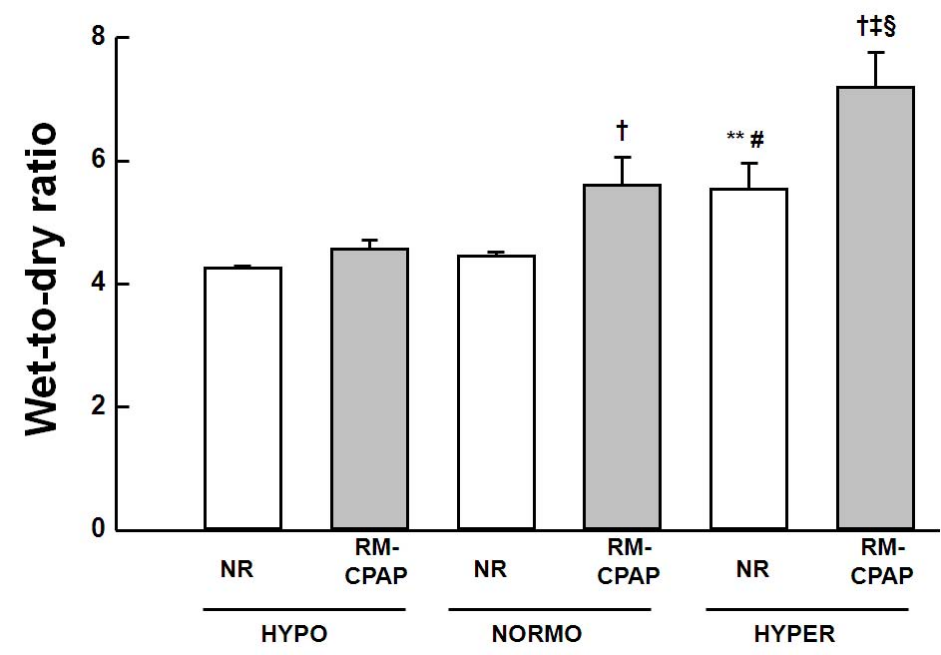
**Wet-to-dry ratio measured after one hour of mechanical ventilation**. Animals were randomly assigned to hypovolemia (HYPO), normovolemia (NORMO) or hypervolemia (HYPER) with recruitment maneuver (RM-CPAP) or not (NR). Values are mean ± standard error of the mean of six rats in each group. †Significantly different from NR (*P *< 0.05). **Significantly different from HYPO-NR (*P *< 0.05). ‡Significantly different from HYPO-RM-CPAP (*P *< 0.05). #Significantly different from NORMO-NR (*P *< 0.05). §Significantly different from NORMO-RM-CPAP (*P *< 0.05).

Lung W/D ratio was higher in HYPER than in HYPO and NORMO groups. Furthermore, lung W/D ratio was increased in NORMO and HYPER groups after RMs (Figure 3).

In the NR groups, lung W/D ratio was positively correlated with the fraction area of alveolar collapse (*r *= 0.906, *P *< 0.001) and Est,L (*r *= 0.695, *P *< 0.001), and negatively correlated with PaO_2 _(*r *= -0.752, *P *< 0.001). Furthermore, the fraction area of alveolar collapse was positively correlated with Est,L (*r *= 0.681, *P *< 0.001) and negatively correlated with PaO_2 _(*r *= -0.798, *P *< 0.001). In the RM-CPAP groups, lung W/D ratio was positively correlated with the fraction area of alveolar collapse (*r *= 0.862, *P *< 0.001) and Est,L (*r *= 0.704, *P *< 0.001), while there was no correlation with PaO_2_. In addition, the fraction area of alveolar collapse was positively correlated with Est,L (*r *= 0.803, *P *< 0.001), but not with PaO_2_.

**Figure 4:**
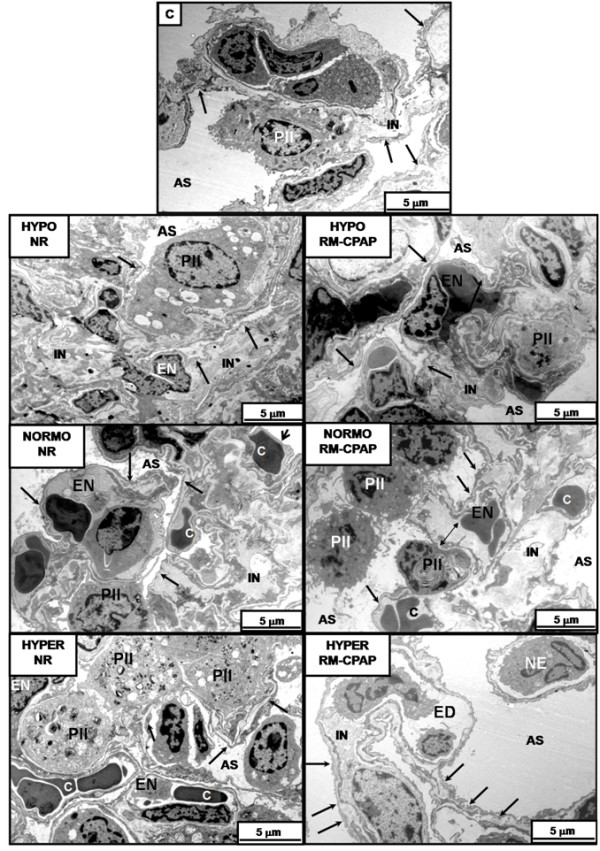
**Electron microscopy of lung parenchyma**. Animals were randomly assigned to hypovolemia (HYPO), normovolemia (NORMO) or hypervolemia (HYPER) with recruitment maneuver (RM-CPAP) or not (NR). Type II pneumocyte (PII) as well as alveolar capillary membrane were damaged in all acute lung injury groups. Note that the alveolar-capillary membrane is less damaged in the HYPO-RM-CPAP group (ellipse) compared with the other groups. In NORMO-RM-CPAP, there was a detachment of alveolar capillary membrane (arrow). In HYPER-RM-CPAP, note that alveolar compartmentalization is lost with disorganization of the alveolar cellular components. Photomicrographs are representative of data obtained from lung sections derived from six animals. EN, endothelial cell.

Figure 4 depicts typical electron microscopy findings in each group. ALI animals showed injury of cytoplasmic organelles in type II pneumocytes (PII) and aberrant lamellar bodies, as well as endothelial cell and neutrophil apoptosis. Detachment of the alveolar-capillary membrane and endothelial cell injury were more pronounced in HYPER compared with HYPO and NORMO groups (Table 4). When RMs were applied, hypervolemia resulted in increased detachment of the alveolar capillary membrane, as well as injury of PII and endothelium, compared with normovolemia.

**Table 4:** Semiquantitative analysis of electron microscopy

	HYPO		NORMO		HYPER	
			
	NR	RM-CPAP	NR	RM-CPAP	NR	RM-CPAP
Alveolar capillary membrane	2 (2-2.5)	2 (2-3)	2 (2-2.25)	3 (2-3)	3**# (3-3.25)	4‡§ (3.75-4)

Type II epithelial cell	2 (2-2.25)	3 (2-3)	2 (2-2.25)	3 (2-3)	3 (2.75-4)	4‡§ (3.75-4)

Endothelial cell	2 (1.75-2.25)	2 (2-3)	2 (2-2.25)	3 (2.75-3)	3**# (3-4)	4‡§ (3.75-4)

Pathologic findings were graded according to a five-point semi-quantitative severity-based scoring system: 0 = normal lung parenchyma, 1 = changes in 1 to 25%, 2 = 26 to 50%, 3 = 51 to 75%, and 4 = 76 to 100% of the examined tissue. Animals were randomly assigned to hypovolemia (HYPO), normovolemia (NORMO) or hypervolemia (HYPER) with recruitment maneuver (RM-CPAP) or not (NR). Values are the median (25^th ^percentile to 75^th ^percentile) of five animals per group. **Significantly different from HYPO-NR (*P *< 0.05). ‡ Significantly different from HYPO-RM-CPAP (*P *< 0.05). #Significantly different from NORMO-NR (*P *< 0.05). §Significantly different from NORMO-RM-CPAP (*P *< 0.05).

**Figure 5:**
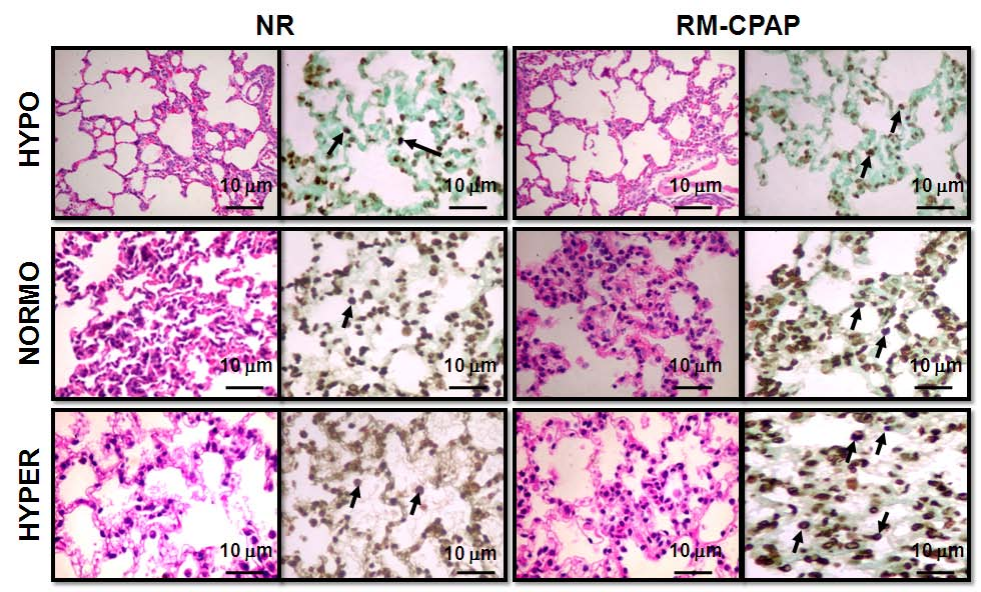
**Representative photomicrographs of lung stained with H&E (left panels) and TUNEL (right panels)**. Animals were randomly assigned to hypovolemia (HYPO), normovolemia (NORMO) or hypervolemia (HYPER) with recruitment maneuver (RM-CPAP) or not (NR). Note that in the HYPER group, the number of apoptotic lung epithelial cells was higher than in NORMO and HYPO (arrows). Photographs were taken at an original magnification of × 200.

Hypervolemia did not increase apoptosis of lung, kidney, liver, and small intestine villous cells (Table 5). In the HYPER group, RMs led to increased TUNEL positive cells (Table 5 and Figure 5), but not of kidney, liver, and small intestine villous cells.

**Table 5:** Cell apoptosis

	HYPO		NORMO		HYPER	
			
	NR	RM-CPAP	NR	RM-CPAP	NR	RM-CPAP
Lung	2 (2-3)	2 (2-2.25)	2 (1.75-3)	2 (2-3)	3 (2-3.25)	4‡§ (3-4)

Kidney	2 (2-3)	3 (2-3.25)	3 (1.75-3)	3 (2-3)	3 (2.75-3.25)	4 (3-4)

Liver	2 (2-2.25)	2 (2-3)	2 (2-3)	2 (2-3)	2 (2-3)	3 (2.75-3.25)

Villi	3 (2-3)	3 (2.75-3.25)	3 (2-3)	3 (2.75-3)	3 (3-4)	4 (2.75-4)

Semi-quantitative analysis of apoptotic cells in lung, kidney, liver, and small intestine villi. The apoptotic findings were graded as negative = 0, slight = 1, moderate = 2, high = 3 and severe = 4 in 10 non-coincident microscopic fields (× 400 magnification). A mean score was then calculated (0 = normal lung parenchyma; 1 = 1-25%; 2 = 26 to 50%; 3 = 51 to 75%; 4 = 76 to 100% of structures altered). Animals were randomly assigned to hypovolemia (HYPO), normovolemia (NORMO) or hypervolemia (HYPER) with recruitment maneuver (RM-CPAP) or not (NR). Values are the median (25^th ^percentile to 75^th ^percentile) of five animals per group. ‡Significantly different from HYPO-RM-CPAP (*P *< 0.05). §Significantly different from NORMO-RM-CPAP (*P *< 0.05).

**Figure 6:**
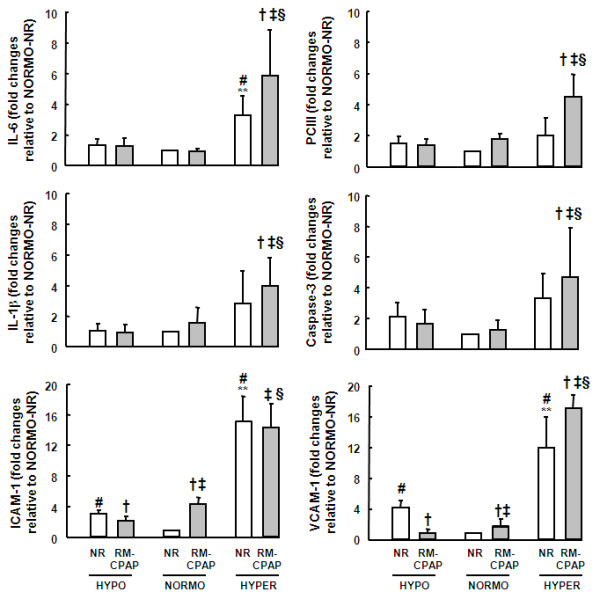
**RT-PCR analysis of caspase-3, IL-6, IL1-β, type III procollagen (PCIII), intercellular adhesion molecule 1 (ICAM-1), and vascular cell adhesion molecule 1 (VCAM-1) mRNA expressions in lung tissue**. Animals were randomly assigned to hypovolemia (HYPO), normovolemia (NORMO) or hypervolemia (HYPER) with recruitment maneuver (RM-CPAP) or not (NR). The y axis represents fold increase compared with NORMO-NR. Values are mean ± standard error of the mean of five animals in each group. †Significantly different from NR (*P *< 0.05). **Significantly different from HYPO-NR (*P *< 0.05). ‡Significantly different from HYPO-RM-CPAP (*P *< 0.05). #Significantly different from NORMO-NR (*P *< 0.05). §Significantly different from NORMO-RM-CPAP (*P *< 0.05).

In NR groups, IL-6, VCAM-1, and ICAM-1 mRNA expressions were higher in HYPER compared with the HYPO and NORMO groups. VCAM-1 and ICAM-1 expressions were also higher in HYPO compared with NORMO, reduced after RMs in HYPO, but augmented in NORMO group. In HYPER group, VCAM-1 expression rose after RMs but ICAM-1 remained unaltered. IL-6, IL-1β, PCIII, and caspase-3 mRNA expressions increased after RMs in HYPER group, but not in NORMO and HYPO groups (Figure 6).

## Discussion

In the present study, we examined the effects of RMs in an experimental sepsis-induced ALI model at different levels of MAP and volemia. We found that: 1) hypervolemia increased lung W/D ratio and alveolar collapse leading to an impairment in oxygenation and Est,L. Furthermore, hypervolemia was associated with alveolar and endothelium damage as well as increased IL-6, VCAM-1 and ICAM-1 mRNA expressions in lung tissue; 2) RMs reduced alveolar collapse regardless of volemic status. In hypervolemic animals, RMs improved oxygenation above the levels observed with the use of PEEP, but were associated with increased lung injury and higher inflammatory and fibrogenic responses; and 3) volemic status associated or not with RMs had no effects on distal organ injury.

### Methodological aspects

To our knowledge, this is the first study investigating the combined effects of RMs and volemic status in sepsis-induced ALI. We used a CLP model of sepsis because it is reproducible and leads to organ injury that is comparable with that observed in human surgical sepsis [28,29].

Volemic status was assessed by echocardiography. It has been shown that echocardiography provides valuable information on preload and cardiac output [30,31]. An inspired oxygen fraction of 0.3 was used throughout the study to minimize possible iatrogenic effects of high inspiratory oxygen concentration on the lung parenchyma [32]. To avoid possible confounding effects of ventilation/perfusion mismatch on the interpretation of the gas-exchange data, inspiratory oxygen fraction was increased to 1.0 just before arterial blood sampling [33]. All animals underwent protective mechanical ventilation to minimize possible interactions between conventional mechanical ventilation, volemic status, and RMs.

The mRNA expressions of IL-6 and IL-1β in lung tissue were determined due to the role of these markers in the pathogenesis of sepsis and ventilator-induced lung injury (VILI) [34]. Although IL-6 has been implicated in the triggering process of sepsis and correlates with its severity [35], IL-1β has been associated with the degree of VILI [32]. On the other hand, mRNA expression of PCIII was determined because it is the first collagen to be remodeled in the development/course of lung fibrogenesis [36], as well as being an early marker of lung parenchyma remodeling [32,37]. We also measured the levels of mRNA expression of caspase-3, because it represents a surrogate parameter for the final step of apoptosis [38]. Finally, the effects of volemic status and RM on mRNA expressions of ICAM-1 and VCAM-1 were determined because these adhesion molecules are involved in the accumulation of neutrophils in the lung tissue, playing a crucial role in the pathogenesis of VILI [39].

### Effects of volemia on lung and distal organ injury

In severe sepsis aggressive fluid resuscitation is recommended [40]. However, in ALI/ARDS the optimal fluid management protocol is yet to be established. Conservative management of ALI/ARDS prescribes that fluid intake be restricted in an attempt to decrease pulmonary edema, shorten the duration of mechanical ventilation, and improve survival. A possible risk of this approach is a decrease in cardiac output and worsening of distal organ function, both of which are reversed with the liberal approach.

Our data show that a hypervolemic status led to increased lung, but not distal organ injury. In fact, hypervolemia was associated with a more pronounced detachment of the alveolar-capillary membrane as well as injury of endothelial cells. On the other hand, fluid restriction did not increase distal organ injury. Different mechanisms could explain the adverse effects of hypervolemia on lung injury: 1) increased hydrostatic pressures; and 2) augmented capillary blood flow and volume.

During hypervolemia, increased pulmonary edema was induced by altered permeability of the alveolar capillary membrane, which is a common finding in sepsis [41], combined with higher hydrostatic pressure. In the presence of pulmonary edema, the increase in hydrostatic pressures along the ventral-dorsal gradient promoted a reduction in normally aerated tissue, contributing to increased stress/strain and cyclic collapse/reopening [42].

Hypervolemic groups were characterized by impaired oxygenation and higher Est,L. The reduction in oxygenation can be attributed to increased edema and atelectasis. The increase in Est,L suggested higher lung stress in aerated lung areas during inflation. In addition, as the same V_T _was applied in all groups and hypervolemia decreased the normally aerated tissue, the strain in the hypervolemic group may be increased. However, even if stress/strain were higher, we did not observe hyperinflation probably because low V_T _and moderate PEEP levels were applied. In this line, cyclic collapse/reopening has also been recognized as a determinant of VILI [43].

Cardiac output, stroke volume, and ejection fraction were increased during hypervolemia. Increased pulmonary perfusion may also directly damage the lungs. In a model of VILI, Lopez-Aguilar and colleagues [44] have shown that the intensity of pulmonary perfusion contributes to the formation of pulmonary edema, adverse distribution of ventilation, and histological damage.

In hypervolemia, we observed an increase in IL-6 mRNA expression in lung tissue, but PCIII mRNA expression did not change, which may be explained by the absence of hyperinflation [12]. Additionally, VCAM-1 and ICAM-1 mRNA expressions were elevated in HYPER group suggesting endothelial activation due to vascular mechanical stretch.

Despite increased lung injury and activation of the inflammatory process, hypervolemia was not associated with increased distal organ injury. Furthermore, hypovolemia and normovolemia did not contribute to distal organ injury, but rather protected the lungs from further damage. Our observation supports the claim that the lungs are particularly sensitive to fluid overload [45]. Lung-borne inflammatory mediators can spill over into the circulation and promote distal organ injury. However, when protective mechanical ventilation is used, decompartmentalization of the inflammatory process is limited [46].

### Interactions between recruitment maneuvers and volemia

The low V_T _and airway pressure concept has been shown to decrease the mortality in ALI/ARDS patients [1]. Given the uncertain benefit of RMs on clinical outcomes, the routine use of RMs in ALI/ARDS patients cannot be recommended at this time. However, RMs have been shown to improve oxygenation without serious adverse events [11]. Furthermore, other papers suggested that RMs may be useful before PEEP setting, after inadvertent disconnection of the patient from the mechanical ventilator or airways aspiration [47]. Finally, RMs have been proposed to further improve respiratory function in ALI/ARDS patients in prone position [48]. Thus, in our opinion, their judicious use in the clinical setting may be justified.

In our animals, RMs reduced alveolar collapse and increased normal aerated tissue independent of the degree of volemia. Along this line, experimental and clinical studies have shown that improvement in lung aeration is associated with better lung mechanics [49-51]. RMs improved oxygenation during hypervolemia, probably because of the higher amount of collapsed lung tissue, which may increase the effectiveness of RMs reversing atelectasis and decreasing intrapulmonary shunt. Gattinoni and colleagues [51] have shown that the beneficial effects of RMs are more pronounced in patients with higher lung weight and atelectasis. The lack of correlation between reduction in atelectasis and oxygenation after RMs in the HYPO and NORMO groups could also be explained by the redistribution of perfusion [52,53]. After RM, Est,L was reduced in HYPO but not in NORMO or HYPER groups. The improvement in Est,L in HYPO group could be explained by alveolar recruitment, whereas the lack of improvement in the other groups may be related to the combination of alveolar recruitment and the increase in interstitial and/or alveolar edema, with consequent increase in specific Est,L.

RMs increase alveolar fluid clearance [8] and aerated tissue, which may lead to reduced lung stretch and inflammatory mediator release [54]. Our data suggest that RMs in the HYPO and NORMO groups did not result in further damage of epithelial and endothelial cells or increased expression of inflammatory and fibrogenic mediators. In addition, RMs induced higher mRNA expression of VCAM-1 in NORMO and HYPER groups, but not of ICAM-1, which was presented higher in HYPER group regardless of RM. Conversely, in HYPO group after RM, the mRNA expression of VCAM-1 and ICAM-1 decreased, probably reflecting reduced shear stress.

RMs transiently increase lung stress [50], probably damaging the alveolar capillary membrane triggering inflammatory and fibrogenic responses [9,12] and impairing net alveolar fluid clearance [8]. However, the potential of RMs to damage the lung is still a matter of debate [11]. In hypervolemia, our results suggest that despite an improvement in functional parameters, RMs are associated with increased detachment of the alveolar capillary membrane, injury of epithelial type II and endothelial cells, as well as an activation of inflammatory and fibrogenetic response. As previously discussed, hypervolemia *per se *may worsen lung injury, especially at the level of the alveolar capillary membrane. Our results suggest that the negative effects of hypervolemia on lung damage are potentiated by increased stress/strain induced by RMs.

The increase in different inflammatory mediators after RMs in hypervolemia cannot be explained by increased atelectasis and/or cyclic opening and closing of collapsed units. In fact, atelectasis was reduced after RMs in hypervolemia. Thus, the increase in gene expression of inflammatory mediators in the lung may have resulted from a single sustained inflation RM.

There are conflicting data on the potential of RMs to decompartmentalize lung inflammation [55,56]. Our results suggest that the combination of RMs with hypervolemia does not result in distal organ injury. Nevertheless, we cannot extrapolate these results to longer periods of ventilation and/or the application of other strategies to recruit the lungs. Theoretically, the inflammatory process could spread to distal organs in the long term. On the other hand, more frequent RMs could accelerate and exacerbate our findings. Also, RMs with pressure profiles different from the sustained inflation, for example gradual increase of airway pressure, could lead to reduced stress and reduce the biological impact of the maneuver. Certainly, this issue deserves further investigation.

We observed greater injury of type II epithelial cells and gene expression of PCIII when lungs were recruited in hypervolemia. Not only are type II cells involved in surfactant production, they are also associated with repairing mechanisms of injured lungs [57]. Re-expansion of collapsed lung units may expose the alveoli to tensile and shear stresses stimulating fibroblasts and macrophages to synthesize collagen fibers [58]. Our results are in accordance with previous reports demonstrating increased procollagen mRNA expression in lungs submitted to high airway pressures [37].

### Limitations

This study has several limitations. Firstly, we used a CLP model of sepsis. Thus, our results cannot be extended to other experimental models of sepsis or directly extrapolated to the clinical scenario. Secondly, the mortality of our sepsis model was relatively high (40%). Thus, we cannot completely exclude that there was a kind of natural bias and a 'sepsis-tolerating' population has been unintentionally selected. However, if hypervolemia was able to produce and potentiate lung damage after RMs in this subgroup, effects would have been even more pronounced in a less 'sepsis-tolerating' population. Thirdly, the observation time was relatively short (one hour), precluding extrapolation of our findings to longer periods of ventilation. The one-hour period was chosen based on our experience with this model and taking the time needed to detect alterations in the proinflammatory and fibrogenetic response of the lungs due to mechanical ventilation in rats [21,59]. As we identified that the proinflammatory response was activated and the alveolocapillary membrane was damaged in the short period, we speculate that the protein levels of the inflammatory cytokines would be higher in the lungs with hypervolemia (specially after RMs) and achieve distal organs due to decompartmentalization if the observation period would have been extended. Fourthly, hypervolemia was achieved by infusion of gelatin. Different results may be observed with other types of colloids or even crystalloids. Finally, the RM was performed as sustained inflation. Recent studies have reported reduced lung injury and fewer adverse hemodynamic effects with other types of RM [12]. However, sustained inflation is the most commonly used RM in clinical practice [11].

## Conclusions

In the present model of sepsis-induced ALI, the use of RMs during hypervolemia reduced alveolar collapse and improved oxygenation and lung mechanics at the expense of alveolar capillary membrane damage, increased edema, and higher gene expression of inflammatory and fibrogenic mediators. Our data suggest that hypervolemia, but not normo- or hypovolemia, may induce and also potentiate lung damage after RMs while not affecting distal organs. Therefore, volemic status should be controlled during RMs, but this hypothesis must be tested in further clinical studies.

## Key messages

• Hypervolemia increased lung W/D ratio and alveolar collapse leading to impairment in oxygenation and Est,L. Furthermore, hypervolemia was associated with alveolar and endothelium damage as well as increased mRNA expression of IL-6, VCAM-1 and ICAM-1 in lung tissue.

• RMs reduced alveolar collapse regardless of volemic status.

• During hypervolemia, RMs improved oxygenation and lung mechanics at the expense of alveolar capillary membrane damage, increased edema, and higher gene expression of inflammatory and fibrogenic mediators. Therefore, hypervolemia, but not normo or hypovolemia, may potentiate lung damage after RMs.

• Volemic status should be controlled and hypervolemia avoided during RMs, but this hypothesis must be tested in further clinical studies.

## Abbreviations

ALI: acute lung injury; ANOVA: analysis of variance; ARDS: acute respiratory distress syndrome; CLP: cecal ligation and puncture; Est,L: static lung elastance; FiO_2_: fraction of inspired oxygen; GAPDH: glyceraldehyde-3-phosphate dehydrogenase; H&E: hematoxylin and eosin; HYPER: hypervolemia; HYPO: hypovolemia; ICAM: intercellular adhesion molecule; IL: interleukin; IVC: inferior vena cava; MAP: mean arterial pressure; NORMO: normovolemia; PII: type II pneumocytes; PaCO_2_: arterial carbon dioxide partial pressure; PaO_2_: arterial oxygen partial pressure; PCIII: type III procollagen; PEEP: positive-end expiratory pressure; pHa: arterial pH; RA: right atrium; RMs: recruitment maneuvers; RR: respiratory rate; RT-PCR: reverse transcription polymerase chain reaction; TUNEL: Terminal deoxynucleotidyl Transferase Biotin-dUTP Nick End Labeling; VCAM: vascular cell adhesion molecule; VILI: ventilator-induced lung injury; V_T_: tidal volume; W/D: wet-to-dry; ZEEP: zero end-expiratory pressure.

## Competing interests

The authors declare that they have no competing interests.

## Authors' contributions

PLS contributed to animal preparation, performance of experimental work, analysis of mechanical and histological data, statistical analysis, and writing of the manuscript. FFC contributed to animal preparation, performance of experimental work, preliminary data analysis, and drafting of the manuscript. LCF contributed to animal preparation, performance of experimental work, analysis of mechanical data, and drafting of the manuscript. GPO contributed to animal preparation, performance of experimental work, and analysis of mechanical and morphometrical data. CSS contributed to animal preparation, performance of experimental work, analysis of mechanical and morphometrical data, and drafting of the manuscript. DSO contributed to analysis of molecular biology data, and drafting of the manuscript. TMG contributed to analysis of molecular biology data, and drafting of the manuscript. NNR contributed to analysis of echocardiography, and drafting of the manuscript. RCG contributed to analysis of echocardiography, and drafting of the manuscript. CSNBG contributed to analysis of histological data, and drafting of the manuscript. MMM contributed to analysis of molecular biology data, and drafting of the manuscript. VLC contributed to analysis of histological data, and drafting of the manuscript. MGA contributed to experimental design, writing of the manuscript, and supervision and overview of entire project. PP contributed to experimental design, writing of the manuscript, and supervision and overview of entire project. PRMR contributed to experimental design, supervision of experimental work, statistical analysis, writing of the manuscript, and supervision and overview of entire project. All authors revised the manuscript and approved its final version.
